# Down-regulation of Krüppel-like factor-4 by microRNA-135a-5p promotes proliferation and metastasis in hepatocellular carcinoma by transforming growth factor-β1

**DOI:** 10.18632/oncotarget.9934

**Published:** 2016-06-10

**Authors:** Shanshan Yao, Chuan Tian, Youcheng Ding, Qingwang Ye, Yong Gao, Ning Yang, Qi Li

**Affiliations:** ^1^ Department of Oncology, Shanghai East Hospital, Tongji University School of Medicine, Shanghai, 200120,China; ^2^ Department of General Surgery, Shanghai East Hospital, Tongji University School of Medicine, Shanghai, 200120,China; ^3^ Department of Liver Surgery, Eastern Hepatobiliary Surgery Hospital, Second Military Medical University, Shanghai, 200438, China; ^4^ Department of Oncology, Shanghai General Hospital, Shanghai Jiao Tong University School of Medicine, Shanghai, 200080, China

**Keywords:** KLF4, miR-135a-5p, TGF-β1, hepatocellular carcinoma

## Abstract

Krüppel-like Factor-4 (KLF4) is a zinc finger transcription factor which plays an important role in cell cycle, proliferation and apoptosis. In Hepatocellular Carcinoma (HCC), the function of KLF4 has been characterized as tumor suppressor. However, the mechanism remains largely unknown. In this study, we demonstrated that TGF-β1 down-regulated KLF4 by activating miR-135a-5p. MiR-135a-5p promoted proliferation and metastasis in HCC cells by direct targeting KLF4 both in vitro and in vivo. In addition, miR-135a-5p expression was up-regulated in clinical HCC tissues, and was inversely correlated with the expression of KLF4. Taken together, our data indicated that TGF-β1 down-regulated KLF4 by activating miR-135a-5p, promoting proliferation and metastasis in HCC.

## INTRODUCTION

Hepatocellular Carcinoma (HCC) is the most common primary liver cancer in adults. The incidence rate of liver cancer fell from fifth to sixth place while the mortality rate rose from third to second in the world and more than 50% of patients who are diagnosed with liver cancer are reported to be Chinese [1]. Recent studies have demonstrated that hepatocarcinogenesis linked with two main pathogenic mechanisms: (1) cirrhosis associated with hepatitis infection (for example, HBV or HCV), toxins (for example, alcohol or aflatoxin) or metabolic influences, and (2) mutations of oncogenes or tumor suppressor genes [2]. The mechanisms are involved in several important cellular signaling pathways, including EGF/EGFR [3], PI3K/AKT/mTOR [4, 5], RAF/MEK/ERK [6], HGF/c-MET [7] and WNT/β-catenin [8]. Besides that, clinical researches suggest that molecularly targeted drugs block critical signaling pathways to treat for advanced HCC. For example, sorafenib (a small-molecule kinase inhibitor) could prolong advanced HCC patients' overall survival (OS) for around 2-3 months [9]. Therefore, it is beneficial for the treatment of HCC to find a new target.

Krüppel-like Factor-4 (KLF4) is a zinc finger transcription factor which plays an important role in cell cycle, proliferation and apoptosis [10–13]. KLF4 has been confirmed as both tumor suppressor and tumor promoter in different types of cancer [14, 15]. In HCC, the function of KLF4 has been characterized as tumor suppressor including: (1) KLF4/VDR signaling pathway may prevent the progress of HCC [10], (2) overexpression of KLF4 inhibits tumor proliferation, invasion and migration in HCC cells and reverts EMT [16], and (3) high expression level of KLF4 is associated with better survival [17]. Additionally, KLF4 activates TGF-β signaling by binding to the TCE of the AT1R promoter in vascular smooth muscle cells [18]. Decreasing KLF4 and increasing SLUG expression is involved in EMT signaling in advanced primary prostate cancer [19].

MicroRNAs (miRNAs) are a kind of endogenous non-coding single-stranded RNA ranging from 17-25 nucleotides that are involved in various biological-signaling pathways [20]. TGF-β regulates miR-206 and miR-29 to control myogenic differentiation [21]. MiR-103 and miR-107 promote metastasis of colorectal cancer by KLF4 and DAPK [22]. These researches prove that miRNAs have an important connection with both KLF4 and TGF-β.

In this study, we used a miRNA library to identify that miR-135a-5p is a regulator of KLF4, and show their potent effects on TGF-β1 signaling pathway in HCC. Moreover, we found that miR-135a-5p expression was increased in clinical HCC tissues, with concomitant low levels of KLF4, suggesting that up-regulation of miR-135a-5p may be involved in hepatocarcinogenesis.

## RESULTS

### Down-regulation of KLF4 by TGF-β1

**Figure 1:**
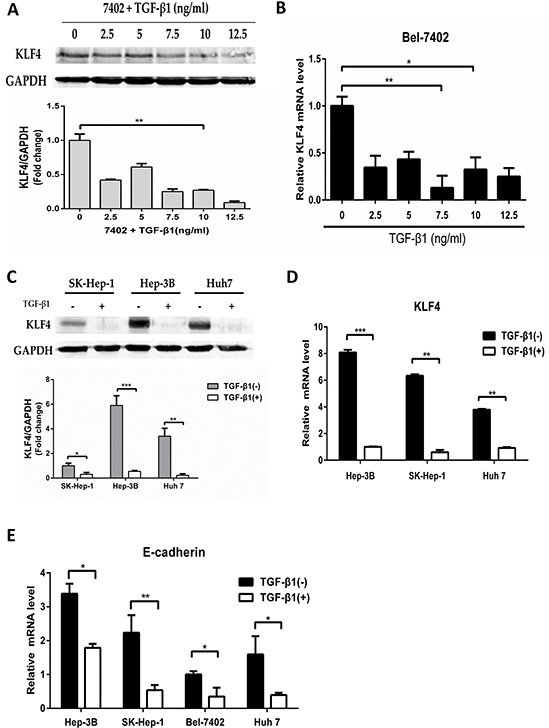
Down-regulation of KLF4 by TGF-β1 **A.** and **B.** Bel-7402 cell was starved for 24h and then treated with TGF-β1 in various concentrations (0-12.5ng/ml) for 24h as indicated. Expression of KLF4 protein level was examined (A) and mRNA level was examined by qRT-PCR (B). The relative mRNA levels were normalized to β-actin and presented as mean ± SEM, with each experiment conducted in triplicate (*, P<0.05; **, P<0.01, n=3). **C.** and **D.** SK-Hep-1, Hep-3B and Huh 7 cells were starved for 24h and then treated with TGF-β1 (10ng/ml) for 24h. The expression of KLF4 protein levels and mRNA levels were examined, respectively (**, P<0.01; ***, P<0.001, n=3). **E.** HCC cells were starved for 24h and then treated with TGF-β1 (10ng/ml) for 24h. The expression of E-cadherin mRNA level was examined by qRT-PCR (*, P<0.05; **, P<0.01, n=3).

Previous studies have indicated that TGF-β induced EMT by both Smad-dependent and Smad-independent pathways which acquire the capacity to detach and migrate away from the primary tumor [23, 24]. Moreover, loss of E-cadherin is considered as a landmark event of EMT that initiates a series of signaling events and major cytoskeletal reorganization [23]. Interestingly, KLF4 is also considered to be associated with EMT, which the expression is reduced during EMT process [19, 25]. Thus, we speculated that TGF-β might regulate the expression of KLF4. To examine this possibility, Bel-7402 was treated with TGF-β1 for various concentrations (0-12.5ng/ml). Activation of the EMT signaling pathway was confirmed by E-cadherin [23]. As expected, the protein level of KLF4 was reduced using TGF-β1 treatment for 24h. Moreover, the higher concentration of TGF-β1 treatment, the more obvious decrease of KLF4 level (Figure 1A). KLF4 mRNA was also decreased after TGF-β1 treatment (Figure 1B). Unlike KLF4, no changes in GAPDH protein or β-actin mRNA levels were observed using TGF-β1 treatment, which justified the use of GAPDH and β-actin as a control. Similar results were observed in other three cells SK-Hep-1, Hep-3B and Huh 7 (Figure 1C and Figure 1D). All these results demonstrated that TGF-β1 repressed the expression of KLF4 in both protein and mRNA levels in various HCC cells.

To examine whether the EMT signaling pathway was activated by TGF-β1, we detected the expression of E-cadherin by qRT-PCR. The results showed that E-cadherin mRNA level was down-regulated more than 60% after TGF-β1 treatment (Figure 1E).

### Up-regulation of MicroRNA-135a-5p by TGF-β1

**Figure 2:**
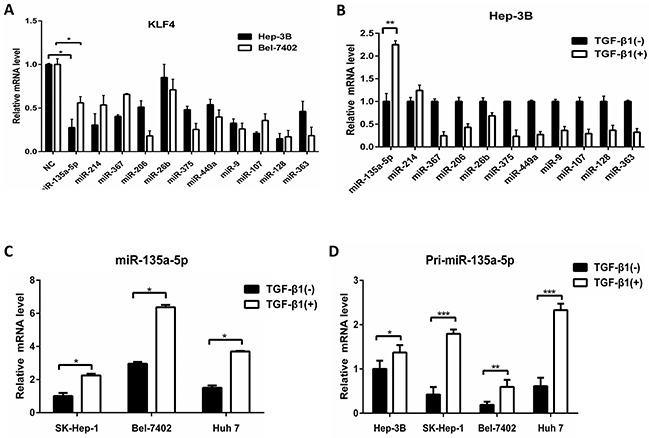
Up-regulation of MicroRNA-135a-5p by TGF-β1 **A.** Hep-3B and Bel-7402 cells were transfected with selected miRNAs mimics at each 100nM concentration (miR-206, miR-26b-5p, miR-214-3p, miR-375, miR-449a, miR-135a-5p, miR-9-5p, miR-107, miR-128-1-5p, miR-363-3p, miR-367), respectively. 48h after transfection, cells were harvested and qRT-PCR analysis of KLF4 normalized to β-actin was performed, data were presented as mean ± SEM (*, P<0.05, n=3). **B.** Hep-3B cell was starved for 24h and then treated with TGF-β1 (10ng/ml) for another 24h. The expression of 11 miRNAs as indicated were measured by qRT-PCR analysis normalized to U6 snRNA (**, P<0.01, n=3). **C.** SK-Hep-1, Hep-3B and Huh 7 cells were starved for 24h and then treated with TGF-β1 (10ng/ml) for another 24h. The expression of miR-135a-5p was measured by qRT-PCR analysis normalized to U6 snRNA (*, P<0.05, n=3). **D.** HCC cells were starved for 24h and then treated with TGF-β1 (10ng/ml) for another 24h. The expression of Pri-miR-135a-5p normalized to β-actin was examined by qRT-PCR (*, P<0.05; **, P<0.01; ***, P<0.001, n=3).

MicroRNAs are known to be involved in various biological-signaling pathways. The reduction of KLF4 expression in response to TGF-β1 treatment suggested that a miRNA could be involved. Thus, we selected 11 miRNAs including miR-206, miR-26b-5p, miR-214-3p, miR-375, miR-449a, miR-135a-5p, miR-9-5p, miR-107, miR-128-1-5p, miR-363-3p, miR-367, which predicted to target KLF4 by binding to regions in the 3′-UTR (Prediction websites: Target Scan Human and MicroRNA. org). So we purchased the 11 miRNAs mimic (Rib Bio, China) as a library to analyze the expression of KLF4 after transfecting them in Hep-3B, the results showed that the expression of KLF4 was almost decreased (Figure 2A). Furthermore, we also tested the expression of the 11 miRNAs after TGF-β1 treatment and only miR-135a-5p was induced at least 1.5-fold (Figure 2B), suggesting that it might be critical for TGF-β1 to mediate reduction of KLF4. To verify the data, we used qRT-PCR to follow the expression of mature miR-135a-5p in other three cells using TGF-β1 treatment, and results were similar (Figure 2C).

Next, we explored the mechanism of miR-135a-5p induction by TGF-β1. miRNAs can be regulated at the level of transcription of the pri-miRNA, or at the Drosha or Dicer processing steps [26].

So we examined whether the expression level of pri-miR-135a-5p is regulated by TGF-β1 in HCC cells. After TGF-β1 treatment for 24h, the level of pri-miR-135a-5p was elevated relative to untreated cells (Figure 2D). Therefore, the results indicated that TGF-β1 is able to enhance the transcription of miR-135a-5p.

### MicroR-135a-5p is critical for down-regulation of KLF4 by TGF-β1

**Figure 3:**
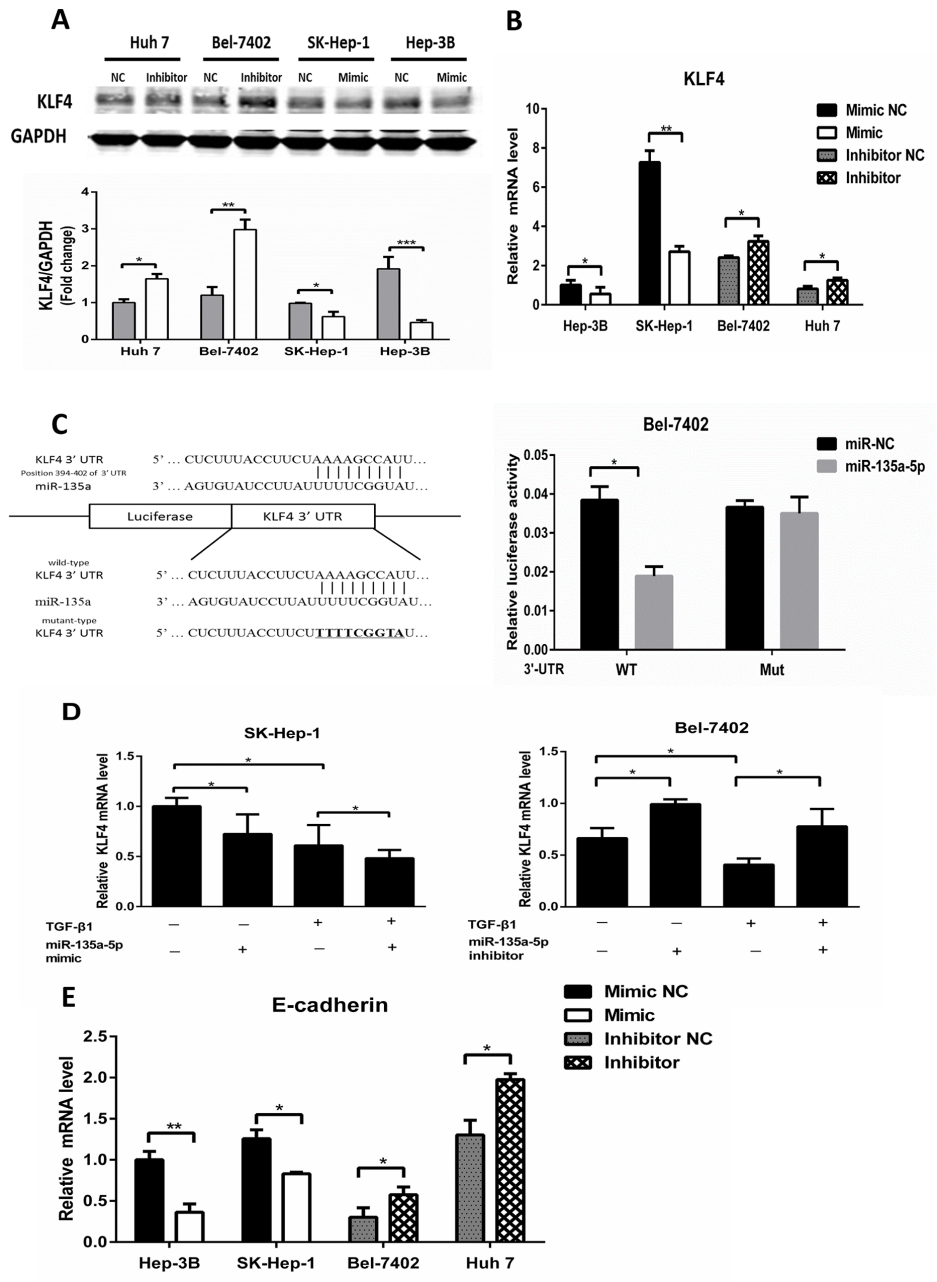
MicroR-135a-5p is critical for down-regulation of KLF4 by TGF-β1 **A.** and **B.** HCC cells were transfected with 100nM miR-135a-5p mimic or 200nM inhibitor, immunoblot and qRT-PCR analysis of KLF4 was performed, data were presented as mean ± SEM (*,P<0.05; **, P<0.01, n=3). **C.** predicted sequences of the miR-135a-5p binding sites within the 3′-UTR of KLF4 and the luciferase reporter constructs containing KLF4 3′-UTR with either wild-type (WT) or mutant(MUT) miR-135a-5p target sites(left). Luciferase activity in Bel-7402 cell upon transfection of miR-135a-5p mimic or NC for 48h together with WT or MUT KLF4 3′-UTR luciferase reporter constructs(*,P<0.05). **D.** SK-Hep-1 and Bel-7402 cell were transfected with miR-135a-5p mimic or inhibitor or their controls for 24h, respectively, and were treated with or without 10ng/mL TGF-β1 for another 24h and then harvested. The mRNA level of KLF4 was examined by qRT-PCR and normalized to β-actin (*, P<0.01, n=3). **E.** HCC cells were transfected with 100nM miR-135a-5p mimic or 200nM inhibitor or their controls, qRT-PCR analysis of E-cadherin was performed (*,P<0.05; **, P<0.01, n=3).

To examine whether miR-135a-5p is able to down-regulate KLF4 in HCC cells, miR-135a-5p mimic and miR-135a-5p inhibitor were transfected into HCC cells, respectively. KLF4 protein and mRNA levels were decreased in miR-135a-5p mimic, and increased in miR-135a-5p inhibitor (Figure 3A and Figure 3B). To further confirm the direct association of miR-135a-5p with the 3′-UTR of KLF4, we predicted the miR-135a-5p binding site in the human KLF4 3′-UTR by TargetScan 6.2, and the 3′-UTR of KLF4 mRNA containing the wild type or mutated putative miR-135a-5p binding sequence was cloned into a luciferase reporter vector, respectively (Figure 3C, left). The luciferase reporter assay demonstrated that miR-135a-5p could effectively inhibit the luciferase activity in the wild type and completely abrogate its regulatory activity in mutation (Figure 3C, right). These results indicated that the level of KLF4 expression was negatively regulated by miR-135a-5p in HCC cells.

To examine the role of miR-135a-5p in TGF-β1 reduced KLF4 expression, miR-135a-5p mimic and miR-135a-5p inhibitor were transfected into SK-Hep-1 and Bel-7402, respectively. Furthermore, both of them were treated with TGF-β1. The data showed that transfected miR-135a-5p mimic, the effect of TGF-β1 on KLF4 was strengthened (Figure 3D, left). However, when transfecting miR-135a-5p inhibitor, the effect of TGF-β1 on KLF4 was abolished (Figure 3D, right).

Next, we detected the expression of E-cadherin in HCC cells, which were transfected miR-135a-5p mimic and miR-135a-5p inhibitor, to examine whether the EMT signaling pathway was activated by miR-135a-5p. The results showed that E-cadherin mRNA level was decreased after transfecting miR-135a-5p mimic, and increased after transfecting miR-135a-5p inhibitor (Figure 3E).

### MicroR-135a-5p promotes proliferation and metastasis in HCC cells by down-regulating KLF4

**Figure 4:**
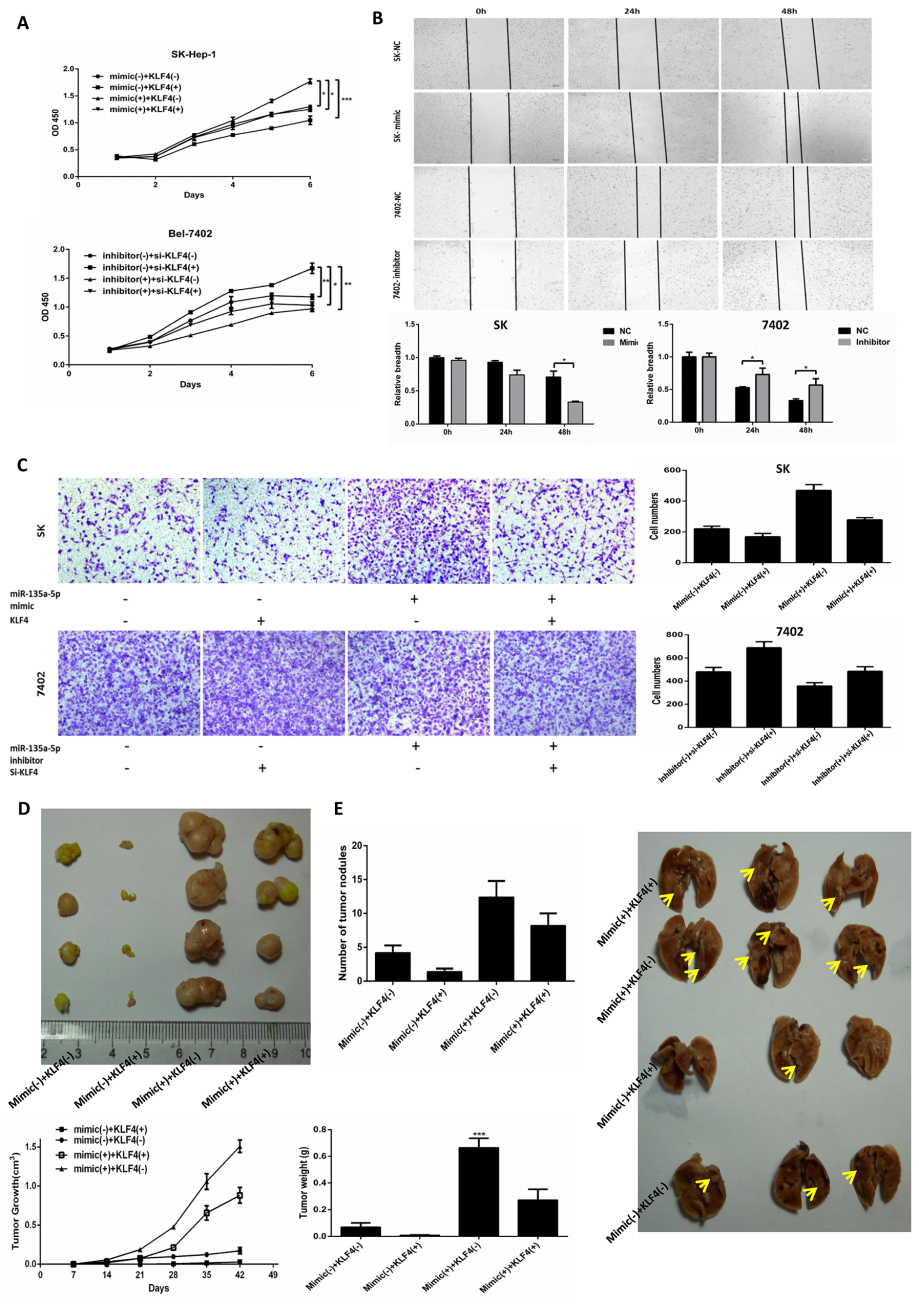
MicroR-135a-5p promotes proliferation and metastasis in HCC cells by down-regulating KLF4 **A.** SK-Hep-1 (upper) cell was transfected with KLF4 or miR-135a-5p mimic or both KLF4 and miR-135a-5p mimic, and Bel-7402 (lower) cell was transfected with si-KLF4 or miR-135a-5p inhibitor or both si-KLF4 and miR-135a-5p inhibitor. The cells were plated into 96-well plates and measured at OD 450nm (*,P<0.05; **, P<0.01; ***, P<0.001, n=3). **B.** SK-Hep-1 (upper) and Bel-7402 (lower) cells were plated in six-well plates overnight and transfected with 100nM miR-135a-5p mimic or 200nM inhibitor or their controls, respectively. Wounds were created by a plastic tip to create several gaps, and the cells were measured by the light microscope at 0h, 24h and 48h (*, P<0.01, n=3). **C.** SK-Hep-1 (upper) cell was transfected with KLF4 or miR-135a-5p mimic or both KLF4 and miR-135a-5p mimic, and Bel-7402 (lower) cell was transfected with si-KLF4 or miR-135a-5p inhibitor or both si-KLF4 and miR-135a-5p inhibitor. Migration assay was photographed after 24h plating. **D.** SK-Hep-1 was divided into four groups (n=5) and infected with miR-135a-5p mimic(−) + KLF4(−), miR-135a-5p mimic(−) + KLF4(+), miR-135a-5p mimic(+) + KLF4(−) and miR-135a-5p mimic(+) + KLF4(+). Nude mice were inoculated subcutaneously with 1×10^6^ cells. Tumors were harvested and photographed after 6 weeks (upper). Moreover, weighed and measured the volume of the tumors at the same time (lower) (n=5). **E.** SK-Hep-1 was divided into four groups (n=5) and infected with miR-135a-5p mimic(−) + KLF4(−), miR-135a-5p mimic(−) + KLF4(+), miR-135a-5p mimic(+) + KLF4(−) and miR-135a-5p mimic(+) + KLF4(+). Nude mice were tail vein injected with 1×10^6^ cells. The lungs were harvested and photographed after 6 weeks (right), and counted the numbers of metastatic nodules on the lung surface (left) (n=5).

To examine whether KLF4 was involved in the miR-135a-5p-mediated tumor-promotive effects in HCC cells, the combinations of transfection were conducted prior to the assessment of cell proliferation and metastasis. In vitro, the data showed that the proliferation was increased in miR-135a-5p mimic(+) + KLF4(−) group and miR-135a-5p mimic(+) + KLF4(+) group compared with miR-135a-5p mimic(−) + KLF4(−) group and miR-135a-5p mimic(−) + KLF4(+) group, respectively (Figure 4A, upper). Furthermore, the proliferation was decreased in miR-135a-5p inhibitor(+) + si-KLF4(−) group and miR-135a-5p inhibitor(+) + si-KLF4(+) group compared with miR-135a-5p inhibitor(−) + si-KLF4(−) group and miR-135a-5p inhibitor(−) + si-KLF4(+) group, respectively(Figure 4A, lower). Next, the wound healing assays showed that miR-135a-5p indeed had the ability to promote proliferation in HCC cells (Figure 4B). Moreover, the cell migration assay was similar with the cell CCK-8 assay (Figure 4C).

To further determine the role of miR-135a-5p and KLF4 in formation of tumor proliferation and metastasis in vivo, male athymic nude mice were randomly divided into four groups and inoculated subcutaneously with 1×10^6^cells in 100μl PBS (miR-135a-5p mimic(−) + KLF4(−), miR-135a-5p mimic(−) + KLF4(+), miR-135a-5p mimic(+) + KLF4(−) and miR-135a-5p mimic(+) + KLF4(+)) at the age of 6 weeks old, respectively. Similarly, mice were tail vein injected with 1×10^6^ cells in 100μl PBS at the same time. After 6 weeks, animals were sacrificed, the tumors and lungs were removed and weighed. We took the photos for the tumors and lungs, and the numbers of metastatic nodules on the lung surface were counted (Figure 4D and Figure 4E). The data showed that the proliferation and metastasis was increased in miR-135a-5p mimic(+) + KLF4(−) group and decreased in miR-135a-5p mimic(−) + KLF4(+) group. All these results demonstrated that miR-135a-5p could promote tumor proliferation and metastasis by down-regulating KLF4 both in vitro and in vivo.

### The relationship between MicroR-135a-5p and KLF4 in HCC patients

**Figure 5:**
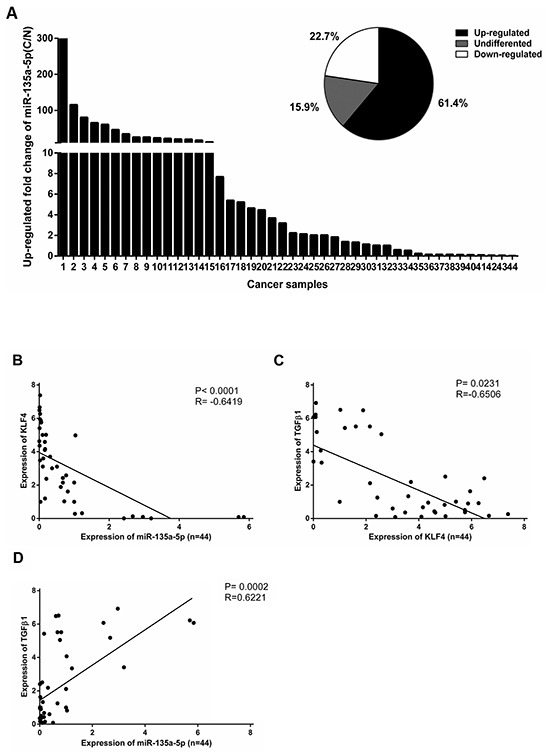
The relationship between MicroR-135a-5p and KLF4 in HCC patients **A.** miR-135a-5p level was examined by qRT-PCR in 44 paired clinical HCC tissues (C) and adjacent non-tumorous tissues (N) and normalized to U6 snRNA. Data were shown as the up-regulated fold change (C/N) of miR-135a-5p level. The percentage of miR-135a-5p expression alteration in 44 paired clinical HCC tissues with “≥1.5” indicating up-regulation, “≤0.5” indicating down-regulation, and “>0.5 but <1.5” indicating no significant. **B.** An inverse correlation between miR-135a-5p and KLF4 expression in 44 paired clinical HCC tissues was measured by linear regression (P<0.0001, R^2^=−0.6419). **C.** An inverse correlation between KLF4 and TGF-β1 expression in 44 paired clinical HCC tissues was measured by linear regression (P= 0.0231, R^2^=−0.6506). **D.** A positive correlation between miR-135a-5p and TGF-β1 expression in 44 paired clinical HCC tissues was measured by linear regression (P= 0.0002, R^2^= 0.6221).

To evaluate the clinical relevance of miR-135a-5p induced down-regulation of KLF4, we analyzed the expression of miR-135a-5p and KLF4 in HCC specimens by qRT-PCR. Patient characteristics and clinical features were observed in Table 1. The results showed that the levels of miR-135a-5p were significantly increased in 27 of 44 clinical HCC tissues relative to the adjacent non-tumorous tissues (Figure 5A). Using linear regression analysis, we found that there was a significant negative correlation between miR-135a-5p and KLF4 expression in HCC tissues (Figure 5B). TGF-β1 and KLF4 had the similar negative correlation (Figure 5C). These results suggested that the relationship between TGF-β1, miR-135a-5p and KLF4 in HCC tissues was consistent with our phenotypic assays in HCC cells.

**Table 1:** Associations between miR-135a-5p expression levels and clinic-pathological features in 44 patients with HCC

	n	miR-135a-5p	P-value
Sex			0.2322
Male	36	0.7405(0.0023-5.8451)	
Female	8	1.3204(0.0369-5.6977)	
Age(years)			0.3584
<50	17	0.9410(0.0369-5.6977)	
≥50	27	0.7861(0.0023-5.8451)	
Tumor size (cm)			0.2793
≤5	31	0.7503(0.0069-5.6977)	
>5	13	1.0739(0.0023-5.8451)	
TNM			0.1338
I+II	10	0.5392(0.0023-2.4197)	
III+IV	34	0.9361(0.0069-5.8451)	
MVI			0.1955
=0	30	0.9446(0.0023-5.8451)	
≥1	14	0.6345(0.0069-3.1961)	

## DISCUSSION

In this study, we demonstrated a critical role and mechanism of TGF-β1 down-regulating KLF4 by activating miR-135a-5p. Moreover, miR-135a-5p promoted proliferation and metastasis as a novel oncogenic miRNA in HCC. In advanced cancer, TGF-β was often overexpressed, and functioned as a promoter by stimulating EMT which strengthens invasiveness and metastasis [27, 28]. TGF-β signaling toward EMT was mediated by both Smad-dependent and Smad-independent pathways, and the Smad pathway was unique to TGF-β signaling [29–31]. Keratinocyte-specific Smad2 ablation resulted in increased epithelial-mesenchymal transition during skin cancer formation and progression [32]. Smad3 ablation in the liver prevented hepatocytic EMT showing that Smad3 was essential for EMT [33, 34]. In addition, E-cadherin expression was induced during TGF-β1-mediated EMT in breast cancer [29]. In this study we demonstrated that E-cadherin expression was decreased after TGF-β1 treatment (Figure 1E), which meant the EMT signaling pathway was exactly activated by TGF-β1.

Previous studies on EMT showed that some transcription factors were controlled by miRNAs. The miR-200 family and miR-205 regulated epithelial to mesenchymal transition by targeting the E-cadherin repressors ZEB1 and ZEB2 [35–38]. Also, TGF-β down-regulated the miR-200 family by inhibiting Akt1 which increased the abundance of ZEB1 and ZEB2 [39]. In addition, the induction of a contractile phenotype in human vascular smooth muscle cells by TGF-β and BMPs was mediated by miR-21 [40]. In this study we developed a miRNA mimic library to identify miR-135a-5p functions, we found that both mature miR-135a-5p and pri-miR-135a-5p levels were increased after TGF-β1 treatment (Figure 2C and Figure 2D), indicating that TGF-β1 could enhance the transcription of miR-135a-5p. Moreover, we detected the expression of E-cadherin after transfected miR-135a-5p mimic and miR-135a-5p inhibitor, and the results showed that the EMT signaling pathway was also activated by miR-135a-5p (Figure 3E). Thus, we speculated that the promoter region of miR-135a-5p might have a direct binding to the related genes in TGF-β signaling pathway, and Smad1/4 or Smad2/3 were the most potential. This speculation needs further validation in the future.

**Figure 6:**
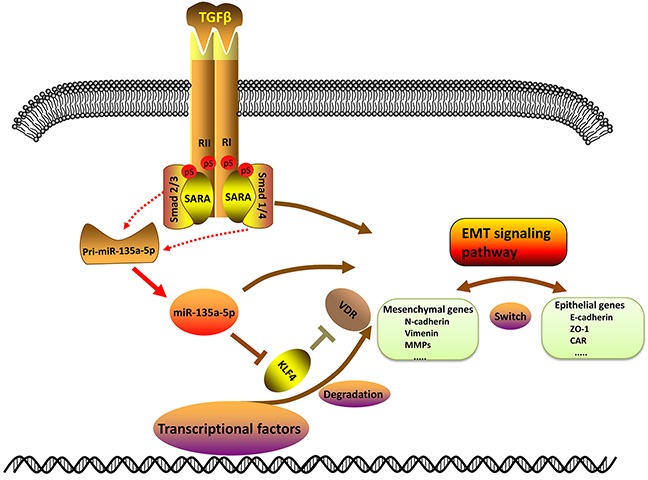
Schematic of TGF-β1 down-regulating KLF4 by activating miR-135a-5p Upon TGF-β1 stimulation, pri-miR-135a-5p expression is able to induce through Smad2/3 or Smad1/4 which is processed into mature miR-135a-5p. Then mature miR-135a-5p represses KLF4 expression by binding to the 3′-UTR of KLF4. And the decrease of KLF4 may further inhibit the expression of VDR. TGF-β1, miR-135a-5p and KLF4 have an impact on the EMT signaling pathway.

KLF4 was a complex transcription factor that can act as a transcriptional activator, a transcriptional repressor, an oncogene, and a tumor suppressor [41]. It was reported that KLF4 negatively regulated EMT of Gastrointestinal Cancers through crosstalk with TGF-β, Notch, and Wnt signaling pathways [25]. In breast cancer, KLF4 inhibits epithelial-to-mesenchymal transition through regulation of E-cadherin gene expression [42]. In this study, we demonstrated the expression of KLF4 after TGF-β1 treatment, the results showed that KLF4 was down-regulated by TGF-β1 at both protein and mRNA level (Figure 1C and Figure 1D). Furthermore, researches prove that miR-7 promotes epithelial cell transformation by targeting KLF4 [43]. MiR-29a promoted colorectal cancer metastasis by regulating matrix metalloproteinase 2 and E-cadherin via KLF4 [44]. Consistent with this idea, in this study, we showed that miR-135a-5p enhanced proliferation and metastasis in HCC by targeting KLF4 both in vitro and in vivo (Figure 4). We also demonstrated that transfected miR-135a-5p mimic in HCC cells, both protein and mRNA levels of KLF4 were significantly decreased (Figure 3A and Figure 3B). Importantly, our results from the luciferase reporter assays showed that miR-135a-5p directly interacted with the KLF4 3′-UTR (Figure 3C). And the relationship among TGF-β1, miR-135a-5p and KLF4 was shown in Figure 3D. Therefore, our findings were consistent in supporting the functional significance of TGF-β1 down-regulating KLF4 by activating miR-135a-5p in hepatocellular carcinoma (Figure 6).

In summary, this study revealed a mechanism of TGF-β1 down-regulating KLF4 by activating miR-135a-5p in HCC. Moreover, miR-135a-5p promoted proliferation and metastasis as a novel “oncomiR” by down-regulating KLF4 whereby directly interacts with KLF4 3′-UTR in HCC. These data suggested that miR-135a-5p and KLF4 should be further explored as potential diagnostic and prognostic markers in hepatocellular carcinoma. We anticipate that this defined loop pathway will provide insight for investigating the regulation of EMT that is proposed to function during tumor progression.

## MATERIALS AND METHODS

### Human tissue specimens

Forty-four HCC tissue specimens were collected from patients who had undergone complete surgical resection at the Department of Hepatic Surgery, the Eastern Hepatobiliary Surgery Hospital of Shanghai Second Military Medical University (Shanghai, China) between March 2015 and July 2015, immediately snap-frozen in liquid nitrogen and stored at −80°C. All tissue specimens (both HCC tissues and adjacent non-cancerous tissues) were constructed for experiments at the same time. Moreover, all patients provided their written informed consent and the hospital ethical committee approved the experiments.

### Cell lines and culture conditions

Four human HCC cell lines SK-Hep-1 (a liver cancer cell), Hep-3B, Bel-7402, Huh 7 were purchased from Shanghai Cell Bank of Chinese Academy of Sciences (Shanghai, China) and cultured in Dulbecco's modified Eagle medium (DMEM, Invitrogen, USA) supplemented with 10% fetal bovine serum (FBS, Hyclone) as well as 100U/ml penicillin and 100μg/ml streptomycin. All of the cells were maintained in a humidified incubator at 37°C with 5% CO2. For TGF-β1 stimulation experiments, 10ng/ml of TGF-β1(R&D systems,USA) was used.

### Plasmids, si-RNA and transfection

The KLF4 expression plasmid was a gift from Dr. Keping Xie (University of Texas MD Anderson Cancer Center, Houston, Texas). Si-KLF4, 5′-AUCGUUGAACUCCUCGGUCUCUCUC-3′ and 5′-G AGAGAGACCGAGGAGUUCAACGAU-3′ (Genepharma, China). For transfection, cells were respectively plated to a density of 70%-80% (plasmid) or 40%-50% (si-KLF4), and all using Lipofectamine 2000 (Invitrogen, USA).

### MiRNA Mimic and miRNA inhibitor

Chemically modified double-stranded RNAs designed to mimic the endogenous mature miR-135a-5p and negative control miRNA were purchased from Genepharma (China). MiRNA mimics were transfected at 100nM concentration using Lipofectamine 2000 (Invitrogen) according to the manufacturer's instructions. Similarly, miR-135a-5p inhibitor and negative control were purchased from Genepharma (China) and transfected at 200nM concentration using Lipofectamine 2000 (Invitrogen, USA) according to the manufacturer's instructions. The sequence of miRNA inhibitor was miR-135a-5p inhibitor: 5′-UCACAUAGGAAUAAAAAGCCAUA-3′; NC inhibitor: 5′-CAGUACUUUUGUGUAGUACAA-3′.

### RNA extraction, cDNA synthesis and quantitative real-time PCR

Total RNA was extracted from cells and tissues by Trizol reagent (Invitrogen, USA) and measured concentration by NanoDrop ND-1000 Spectrophotometer (Agilent, USA). Quantitative real-time PCR (qRT-PCR) was performed to test the expression levels of miR-135a and KLF4 by ABI 7500 System (Applied Biosystems, USA). For quantification of KLF4 mRNA expression, reverse transcription and qRT-PCR amplification were using the PrimeScript RT reagent Kit with gDNA Eraser (Takara, China) and the SYBR Prime Script RT-PCR kit (Takara, Japan). And for miR-135a, reverse transcription and qRT-PCR amplification were using the miDETEC A TrackTM miRNA qRT-PCR Starter Kit (Rib Bio, China). Respectively, β-actin and U6 were measured as an internal control for KLF4, E-cadherin, pri-miR-135a-5p and miR-135a. The primers used in quantitative real-time PCR analysis were designed as follows: For KLF4 5′-GTCTTGAGGAAGTGCTGAGC-3′ (Forward), 5′-AT CGTCTTCCCCTCTTTGGC-3′ (Reverse); For E-cadherin 5′-ATTCTGATTC TGCTGCTCTTG-3′ (Forward),5′-AG TCCTGGTCCTCTTCTCC-3′ (Reverse); For pri-miR-135a-5p 5′-TTGGTCTTGTTTCCCGGTCC-3′ (Forward), 5′-TCACAGCTCCACAGGCTAAC-3′ (Reverse); For β-actin 5′-CCTGGCACCCAGCACAATG-3′ (Forward), 5′-GGGCCGGACTCGTCATACT-3′ (Reverse); For miR-135a-5p 5′-TATGGCTTTTTATTCCTATGTGA-3′ (Reverse); For U6 5′-CTCGCTTCGGCAGCACA-3′ (Forward), 5′-AACGCTTCACGAATTTGCGT-3′ (Reverse). The fold change for the expression levels of each miRNA was calculated using 2-ΔΔCt method. Each experiment was conducted for at least three times.

### Western blotting

The HCC cells were lysed and extracted into protein with 1× SDS–PAGE loading buffer. Protein concentrations were determined using the BCA protein assay kit (Beyotime Institute of Biotechnology, China). Equal amounts of protein were separated on 10% SDS-PAGE gel and then transferred to the PVDF membranes. After blocking with 5% skim milk for 2 hours, the membranes incubated with primary antibodies as follows: KLF4 (1:500, Santa Cruz Biotechnology) and GAPDH (1:3000, Santa Cruz Biotechnology).

### CCK-8 assay

The Cell Counting Kit-8 (CCK-8) assay kit (Dojindo, Japan) was used to test the effect of miR-135a-5p on cell proliferation. Transfected cells were plated into 96-well plates at a density of 2×103 cells per well; 10 μl CCK-8 solution was added to each well the next day, totally six days. The cells were incubated for 70 minutes and the absorbance at 450nm was tested using enzyme-linked immunosorbent assay reader (Dasit, Milan, Italy). Each experiment was conducted for at least three times and the average of the results was analyzed.

### Cell migration assay

The transfected HCC cells in serum-free media were plated into the upper part of a transwell chamber in a 24-well format with 8 mm diameters (Corning, USA) at a suitable density. In the bottom chamber, 800μL of normal DMEM medium containing 10% FBS was added as a chemoattractant and the chambers were incubated for 24-48h at 37°C with 5% CO2. The cells on the upper part were removed by cotton swap and the migrated cells were stained with 0.05% crystal violet for 30 minutes. At last, counting the migrated cells under a microscope in five random fields and each experiment was conducted for at least three times and the average of the results was analyzed.

### Wound healing assay

The transfected HCC cells in DMEM medium containing 10% FBS were plated into six-well plates at a density of 90%. Wounds were created by a plastic tip, cell debris were removed using PBS, and replaced by 0.5% FBS-containing DMEM. The scratched cells were incubated at 37°C with 5% CO2. The initial and residual scratched gap breadth were measured by the light microscope (Nikon, Japan) at 0h, 24h and 48h, respectively. Each experiment was conducted for at least three times.

### Luciferase reporter assay

The 3′-UTR of KLF4 containing an intact miR-135a-5p recognition sequence was cloned into the pmiR-RB-ReportTM (Rib Bio, China). The primer sequences for the wild-type 3′-UTR (959 bp) were: Forward, 5′-GCGCTCGAGCTCGCCTTACACATGAAGA-3′; Reverse, 5′- AATGCGGCCGCAGGAGGAAAACAAAACAAT-3′. For the mutant 3′-UTR, the primer sequences were: Forward, 5′-TACCTTCTTTTTCGGTATATTATGATGTTAGAAGA-3′; Reverse, 5′-TCATAATATACCGAAAAAGAAGGTAAAGAGAATAC-3′. For luciferase assays, cells (5 × 104) were plated in a 24-well plate and incubated for 24h prior to transfection. Firefly luciferase constructs containing the 3′-UTR (or 3′-UTR-mutant) of the potential miR-135a-5p target (100 ng), miRNA mimic, inhibitor or negative control were cotransfected using Lipofectamine 2000 (Invitrogen). Lysates were collected 48h after transfection and measured using a Dual-Luciferase Reporter System (Promega) according to the manufacturer protocol.

### Animal experiments

Male athymic nude mice (4 weeks old) were purchased from Animal Center of the Chinese Academy of Science (Shanghai, China) and maintained in sterile laminar flow cabinets. Mice were randomly divided into four groups and inoculated subcutaneously with 1×10^6^ cells in 100μl PBS at the age of 6 weeks old, respectively. Similarly, mice were tail vein injected with 1×10^6^ cells in 100μl PBS at the same time. After 6 weeks, animals were sacrificed, the tumors and lungs were removed and weighed. The lungs were fixed in formalin overnight before evaluating lung metastasis, and the numbers of metastatic nodules on the lung surface were counted. All animal care and procedures were approved by Tongji University School of Shanghai East Hospital for Animal Experiments.

### Statistical analysis

Statistical analysis was performed using GraphPad Prism 6.0. Differences between two groups were explored by Student's t test. For comparison of paired tissues, the values were presented as mean ± SEM. Only a P value of less than 0.05 was considered significant. “*” indicates P<0.05; “**” indicates P<0.01; “***” indicates P<0.001.

## References

[1] World Cancer Report (2014). http://www.who.int/hinari/news/World_Cancer_Report_2014/en/.

[2] Whittaker S, Marais R, Zhu AX (2010). The role of signaling pathways in the development and treatment of hepatocellular carcinoma. Oncogene.

[3] Huang MJ, Hu RH, Chou CH, Hsu CL, Liu YW, Huang J, Hung JS, Lai IR, Juan HF, Yu SL (2015). Knockdown of GALNT1 suppresses malignant phenotype of hepatocellular carcinoma by suppressing EGFR signaling. Oncotarget.

[4] Simioni C, Cani A, Martelli A, Zauli G, Alameen AA, Ultimo S, Tabellini G, McCubrey JA, Capitani S, Neri L (2015). The novel dual PI3K/mTOR inhibitor NVP-BGT226 displays cytotoxic activity in both normoxic and hypoxic hepatocarcinoma cells. Oncotarget.

[5] Hahn-Windgassen A, Nogueira V, Chen CC, Skeen JE, Sonenberg N, Hay N (2005). Akt Activates the Mammalian Target of Rapamycin by Regulating Cellular ATP Level and AMPK Activity. Journal of Biological Chemistry.

[6] Yoshida T, Hisamoto T, Akiba J, Koga H, Nakamura K, Tokunaga Y, Hanada S, Kumemura H, Maeyama M, Harada M, Ogata H, Yano H, Kojiro M, Ueno T, Yoshimura A, Sata M (2006). Spreds inhibitors of the Ras/ERK signal transduction are dysregulated in human hepatocellular carcinoma and linked to the malignant phenotype of tumors. Oncogene.

[7] Lu S, Török HP, Gallmeier E, Kolligs FT, Rizzani A, Arena S, Göke B, Gerbes AL, De Toni EN (2015). Tivantinib (ARQ 197) affects the apoptotic and proliferative machinery downstream of c-MET: role of Mcl-1 Bcl-xl and Cyclin B1. Oncotarget.

[8] Ma L, Wang X, Jia T, Wei W, Chua MS, So S (2015). Tankyrase inhibitors attenuate WNT/β-catenin signaling and inhibit growth of hepatocellular carcinoma cells. Oncotarget.

[9] Chen J, Gao J (2014). Advances in the study of molecularly targeted agents to treat hepatocellular carcinoma. Drug Discoveries & Therapeutics.

[10] Li Q, Gao Y, Jia Z, Mishra L, Guo K, Li Z, Le X, Wei D, Huang S, Xie K (2012). Dysregulated Krüppel-Like Factor 4 and Vitamin D Receptor Signaling Contribute to Progression of Hepatocellular Carcinoma. Gastroenterology.

[11] Wang B, Zhao MZ, Cui NP, Lin DD, Zhang AY, Qin Y, Liu CY, Yan WT, Shi JH, Chen BP (2015). Krüppel-like factor 4 induces apoptosis and inhibits tumorigenic progression in SK-BR-3 breast cancer cells. FEBS Open Bio.

[12] Yori JL, Seachrist DD, Johnson E, Lozada KL, Abdul-Karim FW, Chodosh LA, Schiemann WP, Keri RA (2011). Krüppel-like Factor 4 Inhibits Tumorigenic Progression and Metastasis in a Mouse Model of Breast Cancer. Neoplasia.

[13] Tian X, Dai S, Sun J, Jin G, Jiang S, Meng F, Li Y, Wu D, Jiang Y (2015). F-box protein FBXO22 mediates polyubiquitination and degradation of KLF4 to promote hepatocellular carcinoma progression. Oncotarget.

[14] Hu D, Wan Y (2011). Regulation of Kruppel-like factor 4 by the anaphase promoting complex pathway is involved in TGF-beta signaling. The Journal of biological chemistry.

[15] Rowland BD, Peeper DS (2006). KLF4 p21 and context-dependent opposing forces in cancer. Nature reviews Cancer.

[16] Lin ZS, Chu HC, Yen YC, Lewis BC, Chen YW (2012). Krüppel-Like Factor 4 a Tumor Suppressor in Hepatocellular Carcinoma Cells Reverts Epithelial Mesenchymal Transition by Suppressing Slug Expression. PLoS ONE.

[17] Hsu HT, Wu PR, Chen CJ, Hsu LS, Yeh CM, Hsing MT, Chiang YS, Lai MT, Yeh KT (2014). High cytoplasmic expression of Kruppel-like factor 4 is an independent prognostic factor of better survival in hepatocellular carcinoma. International journal of molecular sciences.

[18] Zhang XH, Zheng B, Gu C, Fu JR, Wen JK (2012). TGF-beta1 downregulates AT1 receptor expression via PKC-delta-mediated Sp1 dissociation from KLF4 and Smad-mediated PPAR-gamma association with KLF4. Arteriosclerosis thrombosis and vascular biology.

[19] Liu YN, Abou-Kheir W, Yin JJ, Fang L, Hynes P, Casey O, Hu D, Wan Y, Seng V, Sheppard-Tillman H, Martin P, Kelly K (2012). Critical and reciprocal regulation of KLF4 and SLUG in transforming growth factor beta-initiated prostate cancer epithelial-mesenchymal transition. Molecular and cellular biology.

[20] Carleton M, Cleary MA, Linsley PS (2014). MicroRNAs and Cell Cycle Regulation. Cell Cycle.

[21] Winbanks CE, Wang B, Beyer C, Koh P, White L, Kantharidis P, Gregorevic P (2011). TGF-beta regulates miR-206 and miR-29 to control myogenic differentiation through regulation of HDAC4. The Journal of biological chemistry.

[22] Chen HY, Lin YM, Chung HC, Lang YD, Lin CJ, Huang J, Wang WC, Lin FM, Chen Z, Huang HD, Shyy JY, Liang JT, Chen RH (2012). miR-103/107 promote metastasis of colorectal cancer by targeting the metastasis suppressors DAPK and KLF4. Cancer Res.

[23] Xu J, Lamouille S, Derynck R (2009). TGF-beta-induced epithelial to mesenchymal transition. Cell Res.

[24] Fuxe J, Vincent T, Garcia de Herreros A (2014). Transcriptional crosstalk between TGFβ and stem cell pathways in tumor cell invasion: Role of EMT promoting Smad complexes. Cell Cycle.

[25] Cui J, Shi M, Quan M, Xie K (2013). Regulation of EMT by KLF4 in Gastrointestinal Cancer. Curr Cancer Drug Targets.

[26] Davis-Dusenbery BN, Chan MC, Reno KE, Weisman AS, Layne MD, Lagna G, Hata A (2011). down-regulation of Kruppel-like factor-4 (KLF4) by microRNA-143/145 is critical for modulation of vascular smooth muscle cell phenotype by transforming growth factor-beta and bone morphogenetic protein 4. The Journal of biological chemistry.

[27] Heldin CH, Vanlandewijck M, Moustakas A (2012). Regulation of EMT by TGFb in cancer. FEBS Letters.

[28] Jiang Y, Feng X, Zheng L, Li SL, Ge XY, Zhang JG (2015). Thioredoxin 1 mediates TGF-β-induced epithelial-mesenchymal transition in salivary adenoid cystic carcinoma. Oncotarget.

[29] Labelle M, Schnittler HJ, Aust DE, Friedrich K, Baretton G, Vestweber D, Breier G (2008). Vascular endothelial cadherin promotes breast cancer progression via transforming growth factor beta signaling. Cancer Res.

[30] Zhang H, Ma Y, Zhang S, Liu H, He H, Li N, Gong Y, Zhao S, Jiang JD, Shao RG (2015). Involvement of Ras GTPase-activating protein SH3 domain-binding protein 1 in the epithelial-to-mesenchymal transition-induced metastasis of breast cancer cells via the Smad signaling pathway. Oncotarget.

[31] Shan B, Yao TP, Nguyen HT, Zhuo Y, Levy DR, Klingsberg RC, Tao H, Palmer ML, Holder KN, Lasky JA (2008). Requirement of HDAC6 for transforming growth factor-beta1-induced epithelial-mesenchymal transition. The Journal of biological chemistry.

[32] Hoot KE, Lighthall J, Han G, Lu SL, Li A, Ju W, Kulesz-Martin M, Bottinger E, Wang XJ (2008). Keratinocyte-specific Smad2 ablation results in increased epithelial-mesenchymal transition during skin cancer formation and progression. The Journal of Clinical Investigation.

[33] Ju W, Ogawa A, Heyer J, Nierhof D, Yu L, Kucherlapati R, Shafritz DA, Bottinger EP (2006). Deletion of Smad2 in mouse liver reveals novel functions in hepatocyte growth and differentiation. Molecular and cellular biology.

[34] Zavadil J, Cermak L, Soto-Nieves N, Bottinger EP (2004). Integration of TGF- beta/Smad and Jagged1/Notch signalling in epithelial-to mesenchymal transition. The EMBO Journal.

[35] Park SM, Gaur AB, Lengyel E, Peter ME (2008). The miR-200 family determines the epithelial phenotype of cancer cells by targeting the E-cadherin repressors ZEB1 and ZEB2. Genes & Development.

[36] Burk U, Schubert J, Wellner U, Schmalhofer O, Vincan E, Spaderna S, Brabletz T (2008). A reciprocal repression between ZEB1 and members of themiR-200 family promotes EMT and invasion in cancer cells. EMBO reports.

[37] Korpal M, Lee ES, Hu G, Kang Y (2008). The miR-200 Family Inhibits Epithelial-Mesenchymal Transition and Cancer Cell Migration by Direct Targeting of E-cadherin Transcriptional Repressors ZEB1 and ZEB2. Journal of Biological Chemistry.

[38] Gregory PA, Bert AG, Paterson EL, Barry SC, Tsykin A, Farshid G, Vadas MA, Khew-Goodall Y, Goodall GJ (2008). The miR-200 family and miR 205 regulate epithelial to mesenchymal transition by targeting ZEB1 and SIP1. Nature Cell Biology.

[39] Iliopoulos D, Polytarchou C, Hatziapostolou M, Kottakis F, Maroulakou IG, Struhl K, Tsichlis PN (2009). MicroRNAs differentially regulated by Akt isoforms control EMT and stem cell renewal in cancer cells. Science signaling.

[40] Davis BN, Hilyard AC, Lagna G, Hata A (2008). SMAD proteins control DROSHA-mediated microRNA maturation. Nature.

[41] Evans PM, Liu C (2008). Role of Krüppel-like factor 4 in normal homeostasis cancer and stem cells. Acta Biochim Biophys Sin.

[42] Yori JL, Johnson E, Zhou G, Jain MK, Keri RA (2010). Krüppel-like factor 4 inhibits epithelial-to-mesenchymal transition through regulation of E-cadherin gene expression. Journal of Biological Chemistry.

[43] Meza-Sosa KF, Pérez-García EI, Camacho-Concha N, López-Gutiérrez O, Pedraza-Alva G, Pérez-Martínez L (2014). MiR-7 Promotes Epithelial Cell Transformation by Targeting the Tumor Suppressor KLF4. PLoS ONE.

[44] Tang W, Zhu Y, Gao J, Fu J, Liu C, Liu Y, Song C, Zhu S, Leng Y, Wang G, Chen W, Du P, Huang S, Zhou X, Kang J, Cui L (2013). MicroRNA-29a promotes colorectal cancer metastasis by regulating matrix metalloproteinase 2 and E-cadherin via KLF4. British Journal of Cancer.

